# Pediatric Canaliculitis: A Case Report

**DOI:** 10.4274/tjo.galenos.2018.04453

**Published:** 2019-04-30

**Authors:** Elif Eraslan Yusufoğlu, Sabiha Güngör Kobat

**Affiliations:** 1 Elazığ Training and Research Hospital, Ophthalmology Clinic, Elazığ, Turkey

**Keywords:** Canaliculitis, punctoplasty, curettage, Actinomyces

## Abstract

Canaliculitis is a rare disease that relapses when not properly diagnosed and treated. It usually occurs in middle-age and advanced age. It is extremely rare in children and infants. A healthy 12-year-old girl presented with lower eyelid swelling and watery discharge in her right eye. During the last 2 years, the patient had been examined several times for the same complaints but there was no improvement despite treatment. Examination showed that the lower punctum had a pouting punctum appearance, and applying pressure to the lacrimal sac area resulted in purulent discharge. Lavage showed that the lacrimal passage was patent. In light of these clinical findings, the patient was diagnosed with canaliculitis. Punctoplasty with surgical curettage of the dacryoliths were performed. After the surgical procedure, a topical antibiotic was prescribed. Histopathological examination of the dacryoliths revealed that the infective cause was *Actinomyces*. No recurrence or complications were observed during 12 months follow-up.

## Introduction

Canaliculitis is a rare condition caused by infection of the canalicular system by various pathogens. It accounts for about 2% of lacrimal system diseases.^1^

It can be easily diagnosed based on clinical findings; however, because it is rarely encountered by practitioners, patients are often followed long-term for inaccurate diagnoses, resulting in delay of effective treatment. In this article we present a case of primary canaliculitis in a pediatric patient with a long history of misdiagnosis and inappropriate treatment.

## Case Report

**Figure 1:**
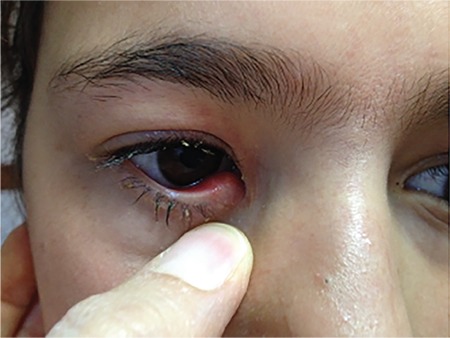
Discharge and swelling in the punctum area of the lower lid of the right eye

A 12-year-old girl presented with a 2-year history of swelling of the medial lower lid and persistent discharge in the right eye. She reported that during this time, she had seen different ophthalmologists and her symptoms had improved slightly with medical treatment, but never completely resolved. Review of her hospital records showed that she had been prescribed various antibiotic and steroid eye drops and ointments for diagnoses of conjunctivitis, chalazion, and lacrimal duct stenosis. External examination of the right eye revealed thick purulent secretion and swelling in the punctum area of the medial lower lid (Figure 1). The lower punctum was enlarged and compression resulted in purulent secretion from the punctum. The lacrimal duct was patent upon irrigation. However, the presence of dacryoliths was felt as the cannula tip was inserted into the lacrimal duct.

**Figure 2:**
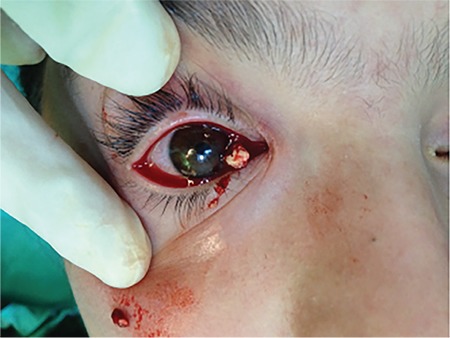
Intraoperative photograph of the patient

**Figure 3:**
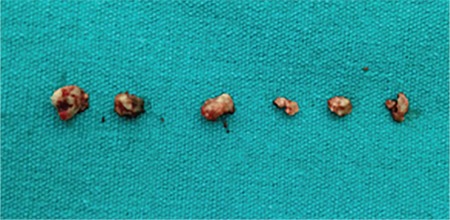
Dacryoliths removed by curettage

**Figure 4:**
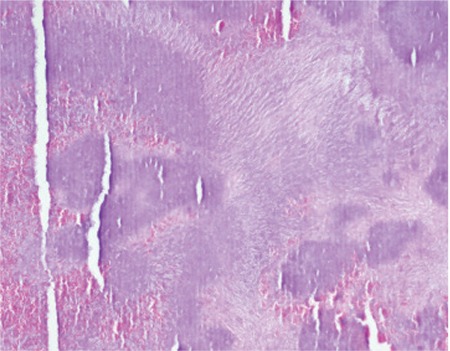
Sulfur granules associated with Actinomyces

Based on the examination findings, the patient was diagnosed with canaliculitis. Due to her history of poor response to long-term medical treatment, we decided to remove the dacryoliths surgically. The patient was admitted for surgery under general anesthesia. We first attempted to spare the canaliculus and remove the dacryoliths by expanding the punctum with a dilator. When this failed to provide a large enough opening, a one-snip punctoplasty was performed. A chalazion curette was used to completely remove the dacryoliths (Figures 2 and 3). The lacrimal system was washed with 5% povidone-iodine solution (Batticon). Postoperatively, the patient was given topical 100,000 U/mL crystallized penicillin 8 times a day for 10 days. The removed dacryoliths were sent for histopathological and microbiological examination. Histopathology revealed sulfur granules associated with *Actinomyces* (Figure 4). Hyphal structures consistent with *Actinomyces* were observed in Gram staining, but culture was negative. At the patient’s last follow-up 12 months later, her symptoms had completely resolved with no recurrence.

## Discussion

Canaliculitis is a suppurative or nonsuppurative infection of the canalicular system and is seen more frequently among women and in the lower canaliculus.^2^ The most common clinical findings are watering, discharge, a pouting punctum appearance, conjunctival redness, and swelling of the canalicular part of the eyelid.^3,4^ Although many cases have been documented in the literature and it is a straightforward clinical diagnosis, it is one of the most misdiagnosed conditions because it is so infrequently encountered.^1^ Patients are often subjected to long-term inappropriate or inadequate treatment for diagnoses such as conjunctivitis, blepharitis, chalazion, mucocele, or dacryocystitis, delaying an accurate diagnosis.^3^ Our patient had also been treated for various diagnoses over a period of 2 years, but had not responded.

Canaliculitis appears at an average age of 60-65 years.^4,5^ However, younger patients have also been reported.^6,7,8^ When seen in infants and children, the clinician should be vigilant for other underlying lacrimal system pathologies.^9,10^ Our patient presented to our center at 12 years of age and her symptoms had started 2 years earlier. There was no accompanying lacrimal system pathology. Therefore, it should be kept in mind that although canaliculitis usually affects older adults, it can also be seen in pediatric patients.

*Actinomyces* spp. are the pathogens most commonly associated with canaliculitis.^11,12,13^ However, the most frequently isolated pathogens in recent microbiological studies are streptococci,^4,14^ staphylococci,^3,15^ or mixed infections.^16^ This difference may be attributable to the difficulty of culturing and microbiologically demonstrating *Actinomyces*. Therefore, histopathological examination of dacryoliths is the best method for demonstrating *Actinomyces*. In fact, this is supported by a report from Ciftci et al.^13^ that although Actinomyces was detected histopathologically in all cases, cultures were positive for only 53.9% of the patients in their study. Similarly, although culture from our patient showed no growth, Actinomyces was detected by histopathology.

Canaliculitis can be treated using a conservative or surgical approach. Conservative treatment employs topical and systemic antibiotics, warm compresses, local massage, and lacrimal system irrigation with antibiotic or iodine-povidone solution. However, resistance and recurrence are common with only topical/systemic antibiotics and massage therapy.^2,15^ This is because dacryoliths and debris in the lacrimal system cause tear stasis and prevent sufficient antibiotic penetration. Lin et al.^4^ treated 25 patients with surgery (canaliculotomy) and 9 patients with medical (local and/or systemic antibiotic) treatment and reported recurrence rates of 33% in the medically treated group and 16% in the surgically treated group. Male sex and the presence of dacryoliths were identified as the main risk factors for recurrence.

In order to increase the effectiveness of conservative therapy and reduce the need for surgery, studies evaluating lacrimal duct irrigation with antibiotics have been conducted. This was intended to enable drugs to better reach the target organism. Mohan et al.^17^ reported improvement in all patients treated with intracanalicular antibiotic irrigation and proposed that this method could provide an alternative to surgery. In another study in which some patients had antibiotic and steroid canalicular irrigation and some underwent surgery, success was achieved in 68% of the irrigated patients and 100% of the surgery group. The authors reported that although surgery was more effective, canalicular irrigation could reduce the need for surgery.^5^

Surgery with topical and/or systemic antibiotic therapy has a high success rate and is considered the gold standard for treatment of canaliculitis. Surgical treatment involves performing curettage after punctum dilatation, punctoplasty, canaliculotomy, or punctum-sparing canaliculotomy. Regardless of which technique is used, complete removal of the canalicular contents is of key importance. Canaliculotomy and punctoplasty enable better removal of canalicular content and generally heal without complications.^2,7,13,18^ However, complications such as narrowing of the canaliculus, lacrimal pump dysfunction, and lacrimal fistulae may occur in rare cases and lead to epiphora in the long term. Kim et al.^19^ reported epiphora in 8.7% of the patients they treated with three-snip punctoplasty and curettage and followed for a mean of 11 months. Due to the risks associated with punctoplasty and canaliculotomy, there has been a recent focus on punctum-sparing surgeries. Buttanri et al.^20^ performed punctum dilation and removed the dacryoliths by applying pressure along the canaliculus from immediately distal to the common canaliculus to the punctum using forceps or a cotton-tipped applicator. Then they completely cleared the canaliculus using a chalazion curette and irrigated with an antibiotic solution. No recurrence was observed in any of the patients during at least 3 months of follow-up. Khu and Mancini^21^ created a horizontal incision along the canaliculus starting 2 mm from the punctum; after removing the dacryoliths, they placed a monocanalicular silicone tube and the incision was left to heal by secondary intention. All of the patients recovered with no complications.

In conclusion, canaliculitis is a recurrent condition that can be easily clinically diagnosed but, because it is encountered so infrequently, is commonly misdiagnosed and inadequately treated for long periods. Although conservative treatment can be effective in some patients, the method is best accepted and has the highest success rate is surgery. The condition is more common in the middle-aged and older age groups. However, it should not be forgotten that, although rare, it can also occur in pediatric patients like ours.
